# Dance at Home for People With Parkinson's During COVID-19 and Beyond: Participation, Perceptions, and Prospects

**DOI:** 10.3389/fneur.2021.678124

**Published:** 2021-05-31

**Authors:** Judith Bek, Michelle Groves, David Leventhal, Ellen Poliakoff

**Affiliations:** ^1^School of Psychology, College of Social Sciences and Law, University College Dublin, Dublin, Ireland; ^2^Division of Neuroscience and Experimental Psychology, School of Biological Sciences, University of Manchester, Manchester, United Kingdom; ^3^Faculty of Education, Royal Academy of Dance, London, United Kingdom; ^4^Mark Morris Dance Group–Dance for PD, Brooklyn, NY, United States

**Keywords:** Parkinson's disease, COVID-19, digital health, dance and movement, home based therapy

## Abstract

Emerging evidence shows that dance can provide both physical and non-physical benefits for people living with Parkinson's disease (PD). The suspension of in-person dance classes during the COVID-19 pandemic necessitated a transition to remote provision via live and recorded digital media. An online survey explored accessibility of and engagement with home-based dance programs, as well as potential benefits and processes involved in participation. The survey was co-developed by researchers and dance program providers, with input from people with PD and physiotherapists. Responses were collected from 276 individuals, including 178 current users of home-based programs, the majority of whom were participating at least once per week. Among respondents not currently using digital resources, lack of knowledge and motivation were the primary barriers. Most participants (94.9%) reported that home based practise provided some benefits, including physical (e.g., balance, posture) and non-physical (e.g., mood, confidence) improvements. Participants valued the convenience and flexibility of digital participation, but noted limitations including reductions in social interaction, support from instructors and peers, and motivation. There was a strong preference (70.8%) for continuing with home-based practise alongside in-person classes in the future. The results indicate that at-home dance is accessible and usable for people with PD, and that some of the previously-reported benefits of dance may be replicated in this context. Digital dance programs will likely remain a key element of future provision for people with PD, and the present findings will inform further development of resources and research into mechanisms and outcomes of home-based dance participation.

## Introduction

Parkinson's disease (PD) is one of the most prevalent and fastest growing neurological conditions (1), and is characterised by multiple motor and sensorimotor symptoms including rigidity, tremor, and disturbances in gait and balance, as well as slower and reduced amplitude movements. PD also causes a range of non-motor symptoms including cognitive, emotional and behavioural problems, pain, and autonomic dysfunction (2).

The coronavirus disease COVID-19 has been associated with a deterioration in PD symptoms (3, 4), and the wider effects of the pandemic may also have exacerbated the social isolation, apathy and anxiety commonly experienced in PD (5, 6). Activities that can mitigate some of these effects by maintaining physical and psychological well-being are therefore likely to be of value.

Non-medical approaches including physiotherapy and exercise are widely recommended for people with PD (7, 8), and dance is a multidimensional activity that offers an engaging, low-cost and sustainable therapeutic option. The popularity of dance as a means of maintaining mobility and health is increasing, and a number of dance programs have been developed specifically for older people and those with neurological conditions, including Silver Swans (Royal Academy of Dance, 2016)^1^ and Dance for PD (Mark Morris Dance Group, 2001)^2^, which feature expanding networks of studio classes delivered by trained instructors worldwide. A substantial body of literature has studied the effects of various dance styles and genres for people with PD, including tango, ballet, modern and mixed styles. Systematic reviews and meta-analyses have reported potential benefits of dance when compared with exercise and social interventions in PD, particularly in terms of motor symptoms, gait, functional mobility and cognition [e.g., (9–12)]. For example, randomised controlled trials of tango dance have shown improvements in balance and functional mobility compared to self-directed exercise (13), and improvements in balance and gait compared to strength and flexibility training (14). Nonetheless, the above meta-analyses have highlighted limitations of studies such as poor quality and risk of bias, and indicated a need for further research to understand effects on non-motor symptoms and quality of life.

When the global spread of COVID-19 in early 2020 necessitated the suspension of group activities such as dance, providers of community dance programs worked quickly to transition to online delivery through video recordings, live streaming and interactive videoconferencing platforms. Recent research has indicated that people with PD were able to use technology-based tools to remain physically active during lockdown (15). However, while the potential value of virtual support groups has been suggested (6), little is known about how people with PD may engage with and benefit from online group activities. Given the emerging evidence showing positive outcomes of dance for people with PD, it is important to understand how they might access and utilise remotely delivered dance classes, and the potential physical and non-physical benefits of these programs.

Beyond maintaining activity and well-being through the COVID-19 pandemic, it is likely that digital classes and resources will change the landscape of community dance in the longer term, for several reasons: (i) restrictions on–or anxiety surrounding–group activities may remain for some time; (ii) a greater volume or variety of digital provision may lead to changes in user preferences, resulting in increased replacement or supplementation of in-person classes; (iii) digital programs could promote broader accessibility and uptake of dance by offering a flexible and convenient alternative, particularly for those who find it difficult to physically attend classes.

Potential limitations of home-based programs should also be considered. For example, the need for confidence to engage with digital technologies, or the ability to afford digital access, may present barriers to participation. Additionally, while at-home dance resources may provide increased participation opportunities for some individuals, others may find it difficult to maintain a routine and motivation. Moreover, the importance of the social aspects of dance in contributing to well-being is often highlighted [e.g., (16–18)], which may be difficult to replicate in virtual and pre-recorded sessions.

Researchers have begun to explore the processes by which dance produces positive outcomes for people with PD. For example, dance involves motor-cognitive processes such as action observation, imitation and imagery, which activate neural networks involved in motor execution (19). Dance programs for PD often incorporate these through exercises such as mirroring and the use of analogy/metaphor images [e.g., (14)], which have been proposed to contribute to beneficial physical and non-physical effects for people with PD (20). Dance also provides other forms of internal and external cues, such as temporal patterning through music, singing and rhythmic counting, that may facilitate movement for people with PD (21–24). These elements may differ between in-person classes and digital participation, which may result in different outcomes. It will also be important to understand whether the format of digital provision (e.g., interactive vs. recorded classes) influences outcomes, and whether at-home dance may be a beneficial activity in itself, or only as an adjunct to in-person classes.

The present study examined experiences and perceptions of digital dance programs among people with PD during the COVID-19 pandemic. The aims were to capture initial data on engagement with these resources, and to identify processes that may be involved, potential benefits and advantages, and limitations of home-based dance. Exploratory analyses also examined the potential influence of frequency and duration of practise, previous participation, digital media type, and engagement with cues and strategies during participation, on the perceived benefits of at-home dance.

## Methods

In a collaboration between researchers and providers of the international community dance programs Dance for PD (Mark Morris Dance Group) and Silver Swans (Royal Academy of Dance), an online survey was designed to collect data from older adults with and without PD who were participating in or interested in home-based dance. To further ensure the relevance of the survey content, input into the design was also obtained from people with PD, dance instructors, and physiotherapists with experience in therapeutic dance settings.

A set of questions was compiled to investigate participants' experiences and views in the following areas: (i) access and usage, (ii) aspects of engagement and experience, (iii) advantages and disadvantages, and (iv) future participation. The majority of questions were fixed-choice to minimise the need for participants to provide lengthy answers. Respondents not currently participating in at-home dance were directed to a subset of questions covering topics such as previous participation, barriers to accessing resources, and potential factors in selecting digital programs for future use.

The survey was created and administered using SelectSurvey.net (v4·033·002; ClassApps, Overland Park, KS, USA) and data collection took place from June to November 2020. The study was approved by the University of Manchester Research Ethics Committee and all participants provided informed consent via an online form, completion of which was mandatory prior to entering the survey.

Potential respondents were contacted through mailing lists and newsletters of dance organisations and community groups. The survey was further advertised through a research volunteer list, social media and direct contact with dance practitioners known to the research team. In this paper we report the fixed-choice responses from people with PD; analysis of the full data set and open comments is ongoing and will be reported separately.

### Data Analysis

Responses to the survey questions are summarised as percentages in Table 1. Statistical analyses were conducted to explore the number of benefits reported in relation to specific aspects of usage and engagement. The effects of duration and frequency of participation and media type used were explored using one-way ANOVAs followed up with Bonferroni-corrected pairwise comparisons. Independent *t*-tests were used to compare the number of perceived benefits according to whether or not respondents had previously attended in-person classes, and according to participants use of cues and strategies during their dance practise (different types of imagery, vocalising, counting and singing).

**Table 1:** Aspects of experience reported (% respondents).

	**Users**	**Non-users**
**Duration of use**		
0–3 months	29.2	
3–6 months	26.4	
6–12 months	18.5	
>12 months	16.9	
**Frequency of use**		
Less than once a week	5.1	
Once a week	29.2	
Twice a week	28.1	
More than twice a week	36.5	
**Type of media/resource**		
Live-streamed class	66.3	
Interactive online class (e.g., Zoom)	47.2	
Pre-recorded online class	69.1	
DVD	28.7	
Other	1.1	
**Preferred format**		
Live (including streamed, interactive)	64.5	
Recorded (including online video, DVD)	18.7	
Both live and recorded	11.2	
No preference	3.7	
**Factors influencing choice of program**		
Dance style (e.g., ballet, modern, mixed)	60.1	51.0
Familiar program/instructor	55.6	18.4
Free classes only	32.0	30.6
Low cost	39.9	43.9
Scheduled classes	53.4	44.9
Opportunities for social connection	30.9	27.6
Reputation/brand	34.3	17.4
Recommendation	37.6	48.0
Difficulty level	41.6	36.7
Type of media/platform	37.6	34.7
**Use of other home-based resources**		
Other home-based activities	77.5	59.2
Online platforms/media for social interaction	73.6	71.4
**Difficulties in accessing or using digital classes**		
Connectivity/network problems	29.2	
Setting up or using software	7.3	
Image quality	6.7	
Sound quality	15.2	
No problems	61.8	
**Elements of engagement**		
Watching the instructor closely	91.6	
Vocalising the movements	23.6	
Singing	42.1	
Counting	44.4	
Imagining how the movements would look if … (visual imagery)	26.4	
Imagining how the movements would feel … (kinesthetic imagery)	18.5	
Imagining moving like something else … (analogy/metaphor imagery)	26.4	
Playing music in the background	4.5	
**Advantages of home-based dance**		
Doesn't require travel	86.0	
Flexible timing	60.7	
Ease of access promotes more frequent participation	54.5	
Maintains privacy	25.3	
Ability to practise at own pace	42.1	
Ability to express oneself without worrying about what others think	34.8	
**Disadvantages of home-based dance**		
Reduced motivation without fixed routine	30.3	
Difficulties in accessing or using technology	14.0	
Lack of suitable space to practise	15.2	
Lack of quiet time to practise	7.3	
Absence of one-to-one support/tuition	25.3	
Absence of social interaction	70.8	
**Aspects missed from in-person participation**		
Interaction with the instructor	63.5	34.7
Interaction with others	70.8	42.9
Support/encouragement	38.8	33.7
Live music	31.5	13.3
Social activities before/after class	40.5	20.4

*Data represent percentages of participants providing a response to each item (i.e., excluding non-respondents)*.

## Results

Responses were collected from 276 individuals with PD, primarily from the United Kingdom (68.1%) and USA (26.4%), with smaller numbers from Canada, Ireland, Switzerland and South Africa. Of these, 178 (75.8% female; mean age 69.5, range 47–88 years) were already using home-based dance programs, while 98 (73.5% female; mean age 67.8, range 45–85 years) were not. Previous attendance at dance class was reported by 78.7% of current users and 50% of non-users.

### Access and Usage

Respondents had been using home-based dance programs for periods ranging from a few weeks to several years, although 77% had started recently as a result of COVID-19 restrictions. The majority were also using online platforms for other forms of exercise and leisure activities, as well as tools for social interaction such as social media sites, messaging applications and video calls.

Most participants (93.8%) were participating at least once a week, with the largest proportion (36.5%) practising more than twice weekly. Participants reported using a variety of media and the majority had used more than one type of resource. Of those using both live (streamed or interactive) and pre-recorded media, 64.5% expressed a preference for live classes. Several different factors were rated as important when choosing classes, with the most frequently endorsed features (>50% of respondents) being dance style, familiarity of program/instructor and provision of scheduled classes.

The most commonly reported type of difficulty in accessing and using resources was connectivity or network problems, with smaller numbers reporting other issues. However, 61.8% of respondents did not experience any problems.

### Aspects of Engagement and Experience

Most participants reported watching the instructor closely during classes, and auditory/rhythmic cues to support movement were engaged through counting, singing and vocalising the movements. Participants also reported using different types of imagery: visual (imagining the look of the movement), kinesthetic (imagining the feeling of the movement), and analogy/metaphor (imagining moving like something else such as an animal, a tree, waves on the ocean or falling leaves).

**Figure 1:**
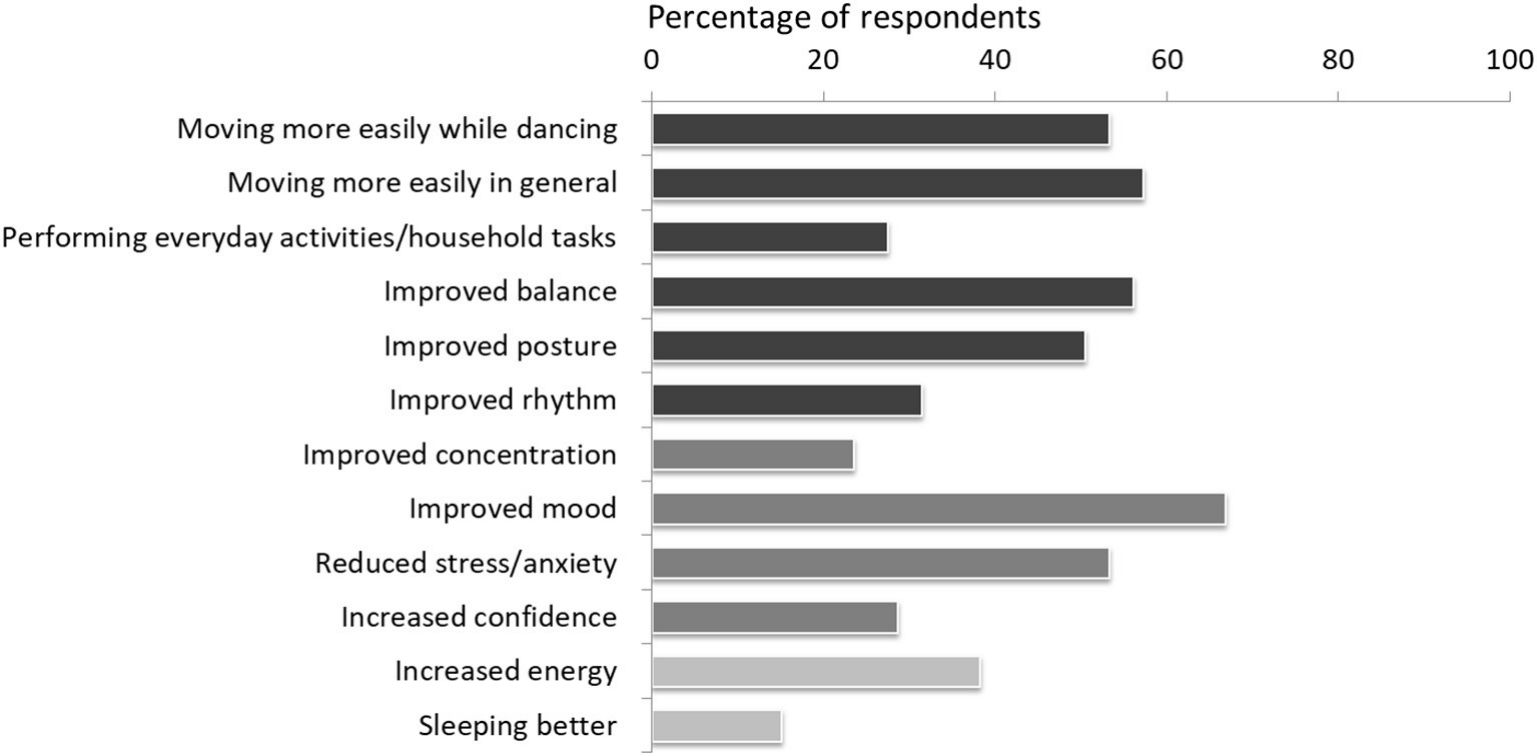
Perceived benefits of home-based dance illustrated by percentage of participants endorsing each outcome. Sensorimotor and functional benefits are indicated by the darker bars, followed by non-motor benefits.

Perceived benefits of engaging with digital programs are presented in Figure 1. These related to sensorimotor and functional abilities as well as cognitive/affective benefits, and improvements in energy and sleep quality. Very few participants (5.1%) did not report any benefits.

### Advantages and Disadvantages

Key advantages of home-based participation were not having to travel to a venue, choice of when to practise, and increased opportunities for frequent participation. The most frequently reported disadvantages were loss of social interaction, reduced motivation without a fixed routine, and absence of one-to-one support and tuition. Among those previously attending in-person classes, participants primarily missed the interactions with other dancers and the instructor.

### Future Participation

**Figure 2:**
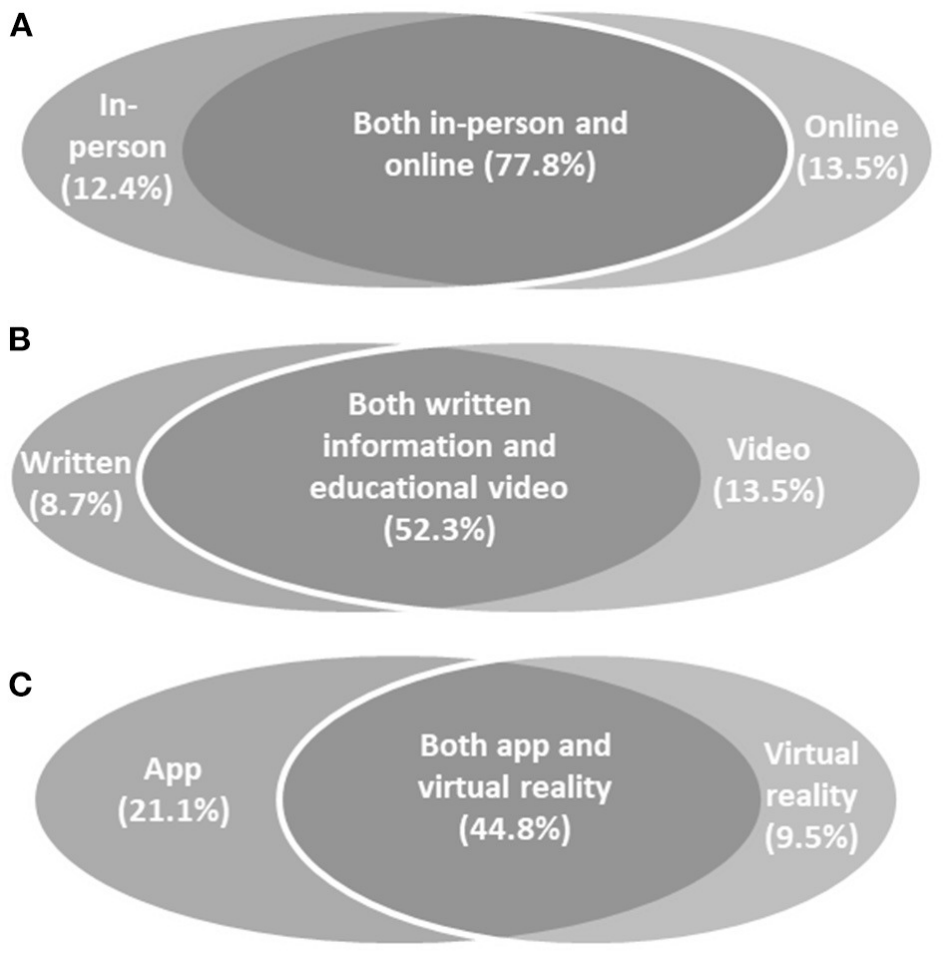
Preferences for future participation: results indicated that the majority of respondents were interested in **(A)** continuing with both in-person and online dance classes, **(B)** receiving educational resources to optimise their dance practise, and **(C)** trying new modes of dance participation such as apps and virtual reality.

Responses to questions about future participation in dance and different modes of delivery are illustrated in Figure 2. The greatest proportion of respondents expressed a preference to continue with both in-person and digital classes, rather than only one mode of participation. The majority expressed an interest in receiving supplementary written and/or video-based educational resources to optimise the benefits of their dance practise, and were open to trying new digital tools for dance such as apps and/or virtual reality.

### Non-users

Among respondents not currently using at-home dance programs, the primary barriers to participation were lack of knowledge about how to access resources (33.9%) and low motivation (30.4%), with a small number of respondents citing lack of time (8.7%), lack of interest (0.9%), not having access to the internet or software required (0.9%), or not expecting to benefit (0.9%). As illustrated in Table 1, factors identified as important in choosing digital programs to use in future reflected those reported by current users, except that “familiar program/instructor” and “reputation/brand” were rated as less important.

### Relationships Between Participation, Media Type, Elements, and Benefits

There was a significant effect of duration of home-based practise on the number of perceived benefits [*F*_(3,155)_ = 4.96; *p* = 0.003]. Respondents using resources for more than 12 months reported more benefits than those participating for 0–3 months (mean difference = 2.04; *p* = 0.024), 3–6 months (mean difference = 2.41; *p* = 0.006) or 6–12 months (mean difference = 2.62; *p* = 0.005). There was also a significant effect of frequency of practise on the number of perceived benefits [*F*_(3,167)_ = 3.79; *p* = 0.012], which was greater among those practising more than twice a week than those practising once a week (mean difference = 1.77; *p* = 0.014). However, there was no difference in the number of perceived benefits according to whether or not participants had previously attended in-person classes [*t*_(169)_ = 0.059; *p* = 0.95].

Media type significantly affected the number of perceived benefits [*F*_(2,166)_ = 3.51; *p* = 0.032]: respondents using a combination of live and pre-recorded classes reported more benefits than those only using pre-recorded materials (mean difference = 1.72; *p* = 0.039).

**Figure 3:**
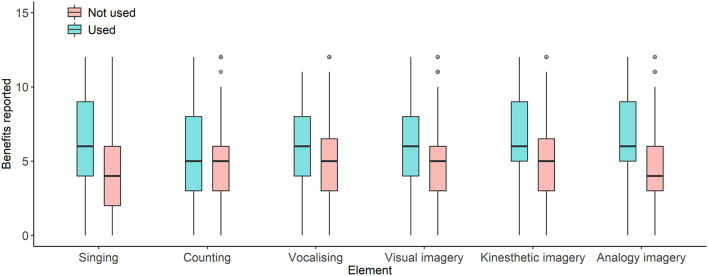
Self-reported benefits in relation to the use of different strategies and cues during home-based dance participation. Boxes show medians with quartiles and dots represent outliers. All three types of imagery (visual, kinesthetic, and analogy/metaphor), as well as singing, were significantly associated with higher numbers of perceived benefits.

As illustrated in Figure 3, greater numbers of benefits were reported by respondents who engaged in visual imagery [*t*_(167)_ = 2.69; *p* = 0.008; *d* = 0.45], kinesthetic imagery [*t*_(167)_ = 2.86; *p* = 0.005; *d* = 0.53], or analogy/metaphor imagery [*t*_(167)_ = 3.93; *p* < 0.001; *d* = 0.66] during classes than participants who did not use these elements. Singing during classes was also associated with a greater number of benefits [*t*_(170)_ = 3.10; *p* = 0.002; *d* = 0.48], but there were no significant differences for counting [*t*_(170)_ = 1.48; *p* = 0.14] or vocalising [*t*_(170)_ = 1.92; *p* = 0.057].

## Discussion

The present study found that people with PD were accessing and using a variety of home-based dance programs and resources during the COVID-19 pandemic, including interactive and streamed live classes, pre-recorded videos and DVDs. Despite concerns about technological barriers, very few participants experienced difficulties in accessing and setting up the required platforms, although connectivity problems were more widely reported. The majority of participants were also using other home-based activity resources and social platforms, consistent with recent reports that people with PD are able to engage in online activities and therapeutic programs (15, 25).

However, around a third of respondents who were not currently using home-based programs reported lacking knowledge of how to find and access them, which may include issues around familiarity with technology. It must also be acknowledged that the present study primarily reached individuals already engaging in digital technologies, with at least sufficient knowledge to use email and complete online surveys. Additionally, the majority of respondents had already participated in dance classes, and so may have been more aware of the availability of online classes and where to access them. Moreover, very few respondents were from outside of the UK and USA. The present findings therefore may not represent the experiences of the wider PD population in accessing digital programs and resources for dance, highlighting the need to raise awareness and support participation among those not currently engaging, and to understand the potential benefits of home-based programs across diverse sections of the PD community.

Most participants had noticed some benefits of their home-based practise, with improvements reported across sensorimotor, functional, cognitive and affective domains, as well as in fatigue levels and sleep. These findings are consistent with reported benefits of in-person participation (10–12, 26–28). Levels of engagement were high, and those practising more frequently reported greater numbers of benefits. Participation for longer than 12 months was also associated with increased benefits, although this may be attributable to previous use of home-based resources alongside in-person classes. Nonetheless, the number of reported benefits was not significantly higher among those previously attending classes, indicating that positive effects may be obtained through home practise alone. Participants using a combination of live and pre-recorded materials reported increased benefits compared to those using pre-recorded videos only.

Given that digital resources are likely to remain an important component of provision going forward, a key finding is that the majority of participants expressed interest in continuing with home practise alongside in-person classes. Transport and logistics have been identified as important factors in determining participation in dance and other therapeutic activities in PD (8, 29), and participants in the present study appreciated the convenience and flexibility of home practise. The high frequency of participation may also reflect this ease of access compared with travelling to classes.

Motivation is critical to engagement with activity programs in people with PD (8, 30), and insufficient motivation was reported as a barrier among those not using digital dance programs in the present study. Current participants also noted reduced motivation as a disadvantage of home-based practise, although this may be somewhat mitigated by providing regularly scheduled classes.

The present findings also reinforce the value of social aspects of dance (17, 18), which can increase motivation to continue (16). The lack of social interaction was the most frequently cited disadvantage of home-based practise and the most commonly missed aspect of in-person classes, followed by the absence of direct support from the instructor. Participants' preference for live classes over pre-recorded videos may reflect the higher level of interaction in this format. Future dance programs could be linked to online peer support groups to provide further opportunities for social interaction, which may increase confidence (29) and help to maintain engagement.

Many participants were interested in receiving educational materials to support their dance practise. This might include guidance on strategies to enhance engagement and optimise the benefits of dance. Respondents were also interested in trying new technologies for dance, such as an app or virtual reality. The feasibility of home-based exercise and dance programs using digital tools has been reported in people with PD [e.g., (31–33)] and, if found to enhance the benefits of dance, such approaches could be integrated into future programs or offered as supplementary options.

Finally, the potential role of motor-cognitive strategies and other cues that may positively impact outcomes of dance such as movement and communication in people with PD [e.g., (20, 23)] was explored in the context of home-based participation. The use of different types of imagery to support movement (visual, kinesthetic and analogy/metaphor), as well as singing, were associated with higher levels of perceived benefits, and future research should examine the significance of these elements in both in-person and digital contexts. For example, imagery may be facilitated by live music and visual input from other dancers' movement in group settings [see (20)], whereas individuals may attend more closely to the instructor's movements, or rely more on internal cues, when participating at home. Further research is also needed to compare the outcomes of in-person and digital dance programs, including different types of home-based practise (e.g., live vs. pre-recorded sessions), and to investigate the longer-term benefits of digital participation.

## Conclusions

The present findings provide initial evidence that home-based dance programs are accessible and usable for people with PD, and may provide similar benefits to in-person programs, although potential barriers to digital participation were identified. There is a clear demand among current participants for continued use of digital resources alongside in-person provision. Furthermore, these resources may also increase the accessibility of dance and its therapeutic effects for people with PD who are unable to attend classes.

## Data Availability Statement

The raw data supporting the conclusions of this article will be made available by the authors, without undue reservation.

## Ethics Statement

The studies involving human participants were reviewed and approved by University of Manchester Research Ethics Committee. The participants provided informed consent via an online consent form.

## Author Contributions

JB: conceptualisation, methodology, investigation, data curation, formal analysis, writing–original draft, writing–review and editing, visualisation, and project administration. MG and DL: methodology, writing–review and editing, and project administration. EP: methodology, writing–review and editing, and visualisation. All authors contributed to the article and approved the submitted version.

## Conflict of Interest

MG is a full-time employee of the Royal Academy of Dance and is involved in the development of its Silver Swans initiative. She has no affiliations with or involvement in any other organisation or entity with any financial interest or non-financial interest in the subject matter or materials discussed in this manuscript. DL is a full-time employee of the Mark Morris Dance Group, which administers the Dance for PD^®^ program. He has no affiliations with or involvement in any other organisation or entity with any financial interest or non-financial interest in the subject matter or materials discussed in this manuscript. The remaining authors declare that the research was conducted in the absence of any commercial or financial relationships that could be construed as a potential conflict of interest.

## Footnotes

^1^https://www.royalacademyofdance.org/classes/silverswans/

^2^https://danceforparkinsons.org/
